# Not All Hereditary Iron Overload Is Hemochromatosis: A Case of Hereditary Xerocytosis Unmasked by Blood Smear Morphology

**DOI:** 10.1002/ajh.27766

**Published:** 2025-07-09

**Authors:** María‐Angustias Molina‐Arrebola, Barbara J. Bain

**Affiliations:** ^1^ Unidad de Hematología y Hemoterapia Área de Biotecnología, Hospital Universitario Poniente El Ejido Almería Spain; ^2^ Centre for Haematology St Mary's Hospital Campus of Imperial College London Faculty of Medicine London UK

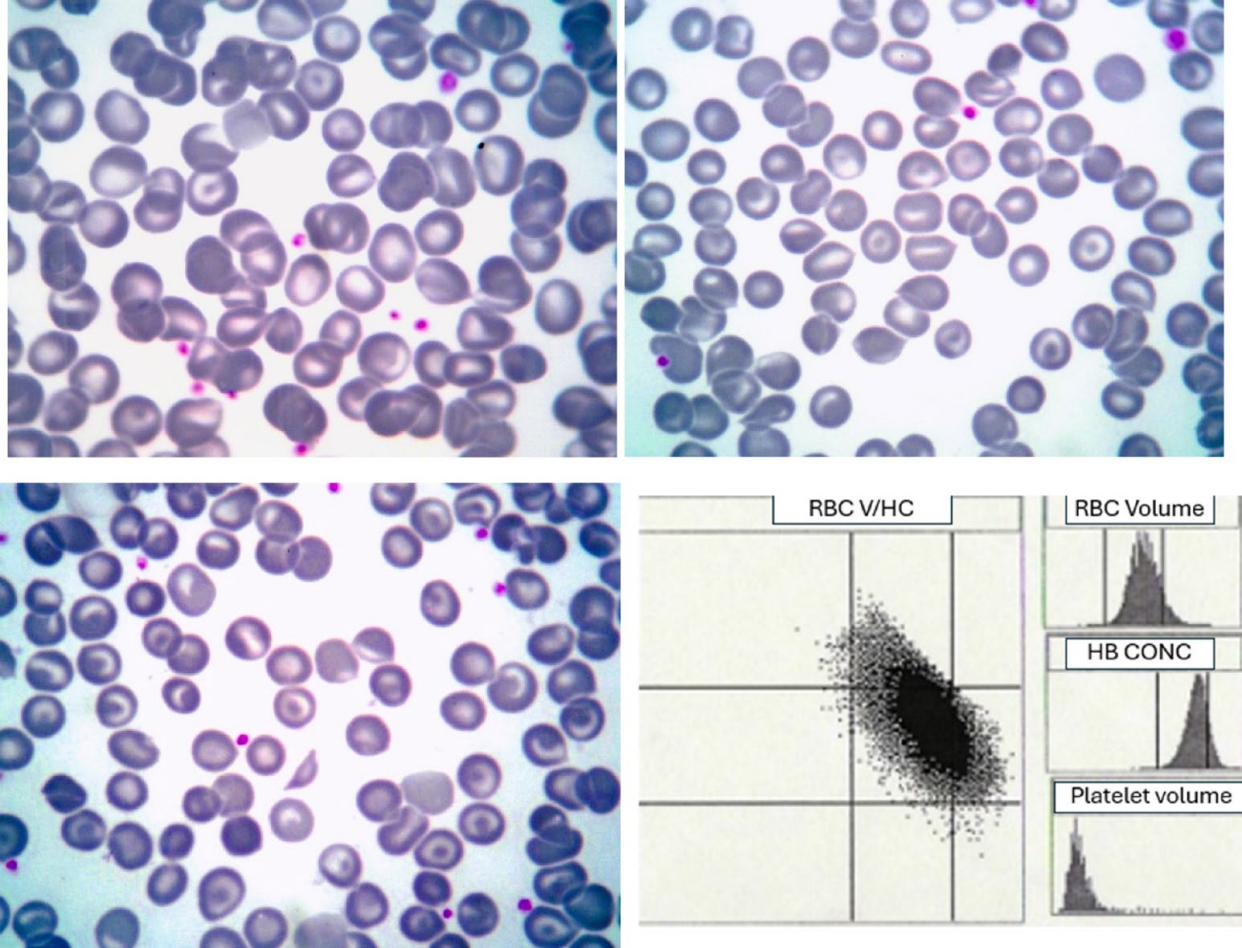

A male patient, now 67‐years‐old, was referred in 1997 for evaluation of suspected hereditary hemochromatosis and for initiation of regular phlebotomy. His blood count showed hemoglobin concentration (Hb) 166 g/L, mean corpuscular volume (MCV) 112.7 fL, mean corpuscular hemoglobin (MCH) 37.8 pg, and mean corpuscular hemoglobin concentration (MCHC) 33.6 g/dL. Platelet and leukocyte counts were normal. Serum iron was 154 μg/dL, transferrin 170 mg/dL, transferrin saturation 90.7%, and ferritin 956.5 ng/mL. Total bilirubin was 1.82 mg/dL with a direct fraction of 0.51 mg/dL. Haptoglobin was 79 mg/dL. Abdominal ultrasonography showed hepatomegaly with a homogeneous liver texture, which was confirmed by computed tomography showing mild parenchymal hyperdensity, likely reflecting increased hepatic iron deposition. Liver biopsy demonstrated grade IV/IV hemosiderosis and septal fibrosis. Based on these findings, a diagnosis of hereditary hemochromatosis and Gilbert syndrome was favored, and regular phlebotomy was commenced. However, genetic testing for common *HFE* mutations (H63D, C282Y, and S65C) was negative, and careful examination of a blood smear (upper images and lower left, May‐Grünwald‐Giemsa, ×100 objective) revealed stomatocytes, knizocytes (stomatocytes with two indentations), irregularly contracted cells, spherocytes, target cells, and occasional schistocytes. The reticulocyte count was consistently elevated (~230 × 10^9^/L). Osmotic fragility testing showed increased red cell resistance. Red cell cytograms showed a marked increase in cells with an increased hemoglobin concentration (lower right image, Advia). Hemoglobin electrophoresis and assays for glucose‐6‐phosphate dehydrogenase and pyruvate kinase were normal.

With later access to molecular diagnostics, the diagnosis of hereditary xerocytosis, suspected from the blood film, was confirmed. A next‐generation sequencing (NGS) panel for hereditary hemolytic anemias revealed the presence of the missense variant c.5975C>T p.(Thr1992Met) in the *PIEZO1* gene, apparently in homozygosity, resulting in the substitution of threonine with methionine at position 1992. The *PIEZO1* gene encodes a component of the Piezo‐type mechanosensitive ion channel. Pathogenic variants in *PIEZO1* are associated with dehydrated hereditary stomatocytosis with or without pseudohyperkalemia and constitutional abnormalities. The variant is listed in the ClinVar database (ID: 3256323) as a variant of uncertain significance (VUS) [1], but our patient demonstrates that it is pathogenic.

Hereditary xerocytosis, also known as the dehydrated form of hereditary stomatocytosis, is a rare inherited hemolytic anemia characterized by increased cation permeability, normal or elevated intracellular cation content, and cellular dehydration [2, 3]. The disorder follows an autosomal dominant inheritance pattern with variable penetrance. It is typically milder than the overhydrated variant. Most cases are associated with a gain‐of‐function mutation in the *PIEZO1* gene. Notably, hepatic iron overload can be disproportionately severe relative to the degree of anemia [3]. Diagnosis is important, both because of the possibility of iron overload and because splenectomy should be avoided.

The patient has continued on regular phlebotomy, and ultrasonography of the liver is now normal.

## Conflicts of Interest

The authors declare no conflicts of interest.

## Data Availability

The authors have nothing to report.
